# Food Allergy: Present and Future Management

**DOI:** 10.1097/WOX.0b013e3181c81fed

**Published:** 2009-12-15

**Authors:** Ananth Thyagarajan, A Wesley Burks

**Affiliations:** 1 The Department of Pediatrics, Division of Allergy and Immunology, Duke University Medical Center, Durham, NC

**Keywords:** food hypersensitivity, treatment, immunotherapy

## Abstract

Food allergy poses a significant burden on patients, families, health care providers, and the medical system. The increased prevalence of food allergy has brought about investigation as to its cause and new treatments. Currently, the only treatment available is to avoid the food and symptomatically treat any reactions. There are multiple clinical and murine models of food allergy treatment that use allergen specific and nonspecific pathways. Allergen specific treatments use mucosal antigen exposure as a method of inducing desensitization and tolerance. Allergen nonspecific methods act via a more global T_H_2 suppressive mechanism and may be useful for those patients with multiple food allergies.

## Introduction

The incidence of atopic disease (asthma, eczema, allergic rhinitis, and food allergy) has significantly increased in Western countries over the last half century,[1] although there is some evidence of its stabilization[2]. The prevalence of food allergy, in particular, has increased[3] that places a large burden on affected patients and their families. It is estimated that food allergy affects 6% of children younger than 3 years old and ~4% of adults[4]. There is no clear reason to this increasing prevalence, but many theories including increased hygiene, increased dietary fat, antioxidants, vitamin D insufficiency, and skin sensitization have been proposed[5]. The impact on the overall medical system is significant. There are an estimated 125,000 emergency room visits related to food allergy in the United States with about 15,000 of these secondary to food induced anaphylaxis[6]. Food allergy not only affects the patient, but the whole family as well. Childhood food allergy has a significant impact on general health perception, emotional impact on the parent, and limitation on family activities[7]. It has also been shown that the diagnosis of food allergy causes significant alterations in meal preparation, social activities, and school attendance and contributed to increased stress levels in the family[8]. The possible mechanisms of food allergy are under investigation and need further elucidation. Alterations in the normal development of tolerance can be the product of a failure to establish oral tolerance or a breakdown in existing tolerance skewing the immune system to a T_H_2 response to these proteins[9,10]. In this article, we will review the current standard of therapy and explore possible future management for food allergy.

## Current Therapy

Currently, the only treatment for food allergy is avoidance of the allergen[11]. Hidden allergens in foods represent a significant problem in manufactured foods. The presence of undeclared allergens in food products represents one of the more common reasons for food product recall in the United States[12]. Of all the food products recalled in 1999, 36% were recalled because they contained one or more undeclared allergens. Although the Food Allergen Labeling and Consumer Protection Act (FALCPA) has been adopted, food packaging and formulation errors, ingredient switching, and foods not covered under this legislation continue to be sources of hidden food allergens[13]. Accidental ingestions also pose a significant threat with events occurring in more than 50% of peanut allergic and in 30% of tree nut allergic children [14].

The majority of food allergy related deaths are secondary to accidental ingestions of peanuts and tree nuts[15]. Reactions secondary to food allergy must be recognized quickly to ensure the timely administration of epinephrine to prevent fatality[11]. Adolescent food allergic patients with comorbid asthma and without access to epinephrine are more likely to have a fatal anaphylaxis reaction[15]. Certain physiologic risk factors (eg, decreased PAF acetylhydrolase activity) have been found that may be used to identify those patients at higher risk for severe anaphylaxis to food [16].

## Primary Prevention

Currently, many international allergy/immunology societies have backed away from recommending long-term dietary restriction during early infancy. In 2008, the American Academy of Pediatrics published its latest statement on early nutritional interventions and their effect on allergy. They found that breast-feeding for at least 4 months can prevent or delay atopic dermatitis, cow's milk allergy, and wheezing in early life. In addition, there was insufficient data to support maternal dietary restrictions during pregnancy and lactation or any dietary intervention beyond 4 to 6 months of age[17].

There is also significant data showing that dietary restrictions may actually increase the risk of atopic disease[18,19]. A major difference of this report versus previous versions is that it does not make recommendations. Instead, statements about possible dietary changes are made along with the presence or absence of its effectiveness. It is important to note that although current evidence does not exist for some of these techniques (ie, any dietary intervention beyond 4 to 6 months of life) that does not necessarily mean that they will prove to be inefficacious with further study [20].

There is some evidence that supplementing breast-feeding with a hydrolyzed formula in at risk infants may prevent some allergic disease[21]. The German Infant Nutritional Intervention (GINI) Study was a prospective trial of a large number of children with high risk of developing atopic disease. These infants were randomized to receive either cow's milk based formula or 1 of 3 hydrolyzed formulas as breast milk supplements for the first 4 months of life. The relative risk of a physician's diagnosis of allergic manifestation compared with cow's milk formula was 0.82 (95% CI, 0.70-0.96) for partially hydrolyzed whey formula, 0.90 (95% CI, 0.78-1.04) for extensively hydrolyzed whey formula, and 0.80 (95% CI, 0.69-0.93) for extensively hydrolyzed casein formula.

The timing of introduction of foods has also been examined as a factor leading to food allergy. Lack et al have shown that the prevalence of peanut allergy in Jewish children from England is 10-fold higher than that of Jewish children in Israel[22]. Because Israeli infants are introduced to peanut during early weaning and continue to eat peanut more frequently and in higher amounts than English infants, this same group questions whether early introduction of peanut during infancy, rather than avoidance, will prevent the development of peanut allergy. They are currently testing this hypothesis through the Learn Early About Peanut Allergy (LEAP) study that is a randomized, controlled trial of infants at high risk of developing peanut allergy (those with eczema and egg allergy) who avoid peanut or eat an age appropriate peanut snack. The primary outcome is to compare the rates of peanut allergy development between the 2 cohorts.

## Allergen Specific Immunotherapy

Potential treatment modalities for food allergy can be categorized as either allergen specific or allergen nonspecific immunotherapy (Table 1). Traditionally allergen specific immunotherapy has focused on antigen delivery to the immune system subcutaneously. Unfortunately, clinical trials of this type of food allergy treatment were shown to be impractical and unsafe[23,24]. Mucosal allergen specific immunotherapy via the sublingual or oral route is accomplished via administering small but increasing doses of food protein in a controlled setting. This is followed by daily home doses of the maximum tolerated amount. An important distinction must be made between desensitization and tolerance. The threshold dose represents the amount of food protein the immune system must encounter to initiate an allergic reaction. Desensitization is the act of increasing the threshold dose to prevent life threatening anaphylaxis. This effect can be short term or prolonged with ongoing therapy. Physiologically this can be accomplished from an increase in food specific IgG4 and a decrease in food specific IgE, this antibody profile can then lead to decreased mast cell and basophil activation and decreased release of inflammatory mediators. It is also possible that desensitization is due, in part, to intrinsic changes in the mast cell and basophil. Tolerance refers to the active adaptation of the immune system via increased Treg development and skewing away from a T_H_2 profile, leading to resolution of the allergy [10].

**Table 1:** Potential Strategies for Treating Food Allergy

Therapy	Mechanism	Status
Allergen specific		
Sublingual immunotherapy	Controlled prolonged exposure to antigen promotes switch from Th2 to Th1 response via promotion of Treg activity	Clinical trials
Oral immunotherapy	Controlled prolonged exposure to antigen promotes switch from Th2 to Th1 response via promotion of Treg activity	Clinical trials
Heat denatured protein	"Natural" immunotherapy by presenting linear but not conformational epitopes to tolerant patients	Clinical trials are on-going
Engineered protein immunotherapy	Mutated IgE-binding epitopes prevent allergic reaction while maintaining T cell activity	Early stage clinical trials
Peptide vaccine	T cell epitope preservation while preventing IgE-cross linking via small overlapping peptides	Murine models
Plasmid DNA encoded vaccines	Tolerance achieved via endogenous production of antigen	Murine models, but strain specific response. No active development
Allergen-conjugated immunostimulatory sequence	Promote Th1 response via activation of innate immune pathway (toll-like receptors)	Clinical trials with allergic rhinitis
Allergen nonspecific		
Monoclonal anti-IgE therapy	Binds and inactivates IgE and prevents stimulation of high affinity IgE-receptor	Monotherapy studies not active, combination studies with other therapies in clinical trials
Chinese herbal medicine	Unknown mechanism, not steroid effect	Early stage clinical trials
Cytokine therapy	Interfere with inflammatory pathways	Clinical trials for eosinophilic esophagitis

### Sublingual Immunotherapy

This therapy uses small amounts of liquid that contain the relevant protein. The drops are held under the tongue for a specified amount of time and then either swallowed or discharged. Enrique et al studied 23 patients with hazelnut allergy confirmed by double-blinded, placebo-controlled food challenges (DBPCFC) who received sublingual immunotherapy (SLIT) with either hazelnut extract or placebo[25]. Of note, almost half of these subjects had symptoms most consistent with oral allergy syndrome. The maintenance treatment delivered 188 *μ*g of Cor a 1 and 122 *μ*g of Cor a 8 to the sublingual mucosa that was held for 3 minutes then discharged. After 8-12 weeks, a subsequent food challenge revealed a significant increase in threshold dose to symptoms in treatment patients versus placebo. The mean hazelnut dose provoking symptoms increased from 2.29 g to 11.56 g in the active group (*P *0.02) versus 3.49 g to 4.14 g in the placebo subjects (not significant). Almost 50% of patients who underwent treatment reached the highest dose (20 g), but only 9% in the placebo. Oral pruritis occurred in 7.4% of all doses, while systemic reactions were observed in only 0.2% of the total doses administered. Mechanistic assays showed an increase in IgG4 and IL-10 levels after immunotherapy in only the active group, but there was no change in the hazelnut-specific IgE. These results provide some evidence for the efficacy of SLIT, but many of the patients had symptoms of oral allergy syndrome. It is unclear whether these findings support SLIT for true food allergy versus oral allergy syndrome.

Currently there are multiple ongoing randomized placebo-controlled studies looking at treating other food allergies with SLIT. An ongoing randomized, placebo controlled study we are conducting at Duke University uses SLIT to treat peanut allergic children. The National Institutes of Health sponsored Consortium of Food Allergy Research (CoFAR) is also conducting a randomized, placebo-controlled study of peanut SLIT in peanut allergic adults. In both of these studies, subjects hold their doses sublingually for 2 minutes and then swallow (vs the Enrique protocol).

### Oral Immunotherapy

This treatment entails oral ingestion of the protein mixed in a food vehicle. Induction of desensitization was demonstrated in the majority of 47 food allergy subjects that underwent 54 oral desensitizing treatments using standardized protocols[26]. Some of the patients had multiple food allergies confirmed by DBPCFC and participated in multiple desensitizations. The majority of patients (83.3%) successfully completed the treatment. During treatment, 51.1% of subjects experienced some mild side-effects, which were controlled by the oral administration of antihistamines or sodium cromolyn. Compared with age and sex matched controls who followed a strict elimination diet, subjects undergoing oral immunotherapy (OIT) had a significant decrease in food-specific IgE and increase in IgG4. These immunologic findings led the authors to hypothesize that oral tolerance may be mediated by the same mechanisms as those involved in traditional immunotherapy that is used for respiratory allergies.

Buchanan et al published a successful OIT protocol for egg allergic children[27]. Seven children with egg allergy diagnosed by an in vitro egg-specific IgE of 7 kU/L or greater (2 kU/L or greater for subjects ≤ 2 years of age) or with a positive allergic reaction to egg within 6 months of beginning the study were recruited. These subjects underwent a 24 month egg OIT protocol involving a modified rush, build-up, and maintenance phases. A maintenance dose of 300 mg was used. During the initial DBPCFC on the day maintenance therapy ended, 4 of the 7 patients tolerated 14.7 g of egg protein. Subjects who passed the first challenge underwent a second DBPCFC after a 3 to 4 month interval without OIT, during which they maintained an egg-restricted diet. Two of these subjects passed the second challenge that demonstrated evidence for tolerance induction, but could also represent the subject's naturally outgrowing egg allergy. At the end of the treatment period egg-specific IgG concentrations increased significantly, although egg-specific IgE concentrations did not change. Symptoms related to dosing were generally mild and required at most treatment with oral diphenhydramine.

Some investigators have questioned whether results like this represent the spontaneous resolution of allergy especially in egg and milk allergic subjects. In a randomized, controlled study, 45 egg or cow's milk allergic patients underwent OIT with 4.5 g of egg or 250 mL (8.5 g) of milk, respectively, or were continued on an elimination diet[28]. All treated subjects experienced side effects during the dose escalation and on maintenance therapy, although the authors considered the great majority of these symptoms as mild. At the follow up food challenge (after 2 months of an elimination diet in the OIT subjects) 9 of 25 children (36%) showed tolerance in the treatment and in the control group, 7 of 20 children (35%) were tolerant. These results suggested that the effect of OIT was comparable to the rate of natural spontaneous resolution of allergy. However, an additional 7 of 25 in the treatment group had some level of partial response that could infer protection from accidental ingestion. This would increase the rate of responders to 64%.

More recently, a randomized, placebo controlled study of milk OIT in milk allergic subjects was conducted that produced different results[29]. Twenty milk allergic subjects confirmed by DBPCFC were randomized to milk OIT or placebo. Dosing included 3 phases: the build-up day (initial dose, 0.4 mg of milk protein; final dose, 50 mg), daily doses with 8 weekly dose increases to a maximum of 500 mg, and continued daily maintenance doses for 3 to 4 months. Nineteen patients, 6 to 17 years of age, completed treatment: 12 in the active group and 7 in the placebo group. One dropped out because of persistent eczema during dose escalation. The subsequent food challenge at the end of the maintenance period showed an active group threshold symptom dose much higher that the placebo group (5,140 mg vs 40 mg, *P *= 0.0003). Among treatment doses versus placebo, there were 45.4% versus 11.2% total reactions, with local symptoms being most common. Interestingly, milk-specific IgE did not change, but milk-specific IgG4 increased significantly. Among the placebo subjects, 6 of 7 elected to receive open label active treatment after the unblinding DBPCFC. In those patients, the milk dose threshold was 40 mg before OIT. After OIT, the median was 8,140 mg (*P *= 0.03). These results provide strong evidence for desensitization for this milk OIT protocol.

Similar results have been seen in an ongoing randomized, placebo controlled study of peanut OIT in peanut allergic children taking place at Duke University and the University of Arkansas for Medical Sciences. Thirty-seven peanut allergic subjects between the ages of 1 to 18 years old have been enrolled in this protocol with a maintenance dose of 4000 mg of peanut protein or placebo. There have been 2 subjects that have failed to reach the maintenance dose. Both of these subjects were initial day desensitization failures where allergic symptoms were not tolerated. After 1 year, all treatment subjects have tolerated 5000 mg of peanut protein during food challenge compared with placebo patients who tolerated a median dose of 460 mg (*P *< 0.001). After 6 months on therapy the mean titrated skin prick test (SPT) and basophil activation have decreased significantly in the treatment group (unpublished data). Mechanistic assays from an earlier open label trial of peanut oral immunotherapy demonstrated that the mean peanut-specific IgE initially increased but then decreased at 12 and 18 months. The peanut-specific IgG4 increased to a peak at 24 months and the number of FoxP3+ Tregs also increased to a peak at 12 months [30].

These initial results suggest that food specific OIT is safe and effective in attaining desensitization. Although desensitization in itself is better than complete avoidance, the induction of tolerance would be the ultimate goal. The ability of these therapies to induce tolerance is currently the focus of ongoing investigations. For a summary of selected SLIT and OIT studies refer to Table 2.

**Table 2:** Selected SLIT and OIT Studies

	Allergen Received (No. of Subjects)	Length of Therapy	Efficacy
Sublingual			
Enrique et al. [25]	Hazelnut (11)	8-12 weeks	Five (45%) of 11 reached highest dose (20 g) in food challenge.
Bird et al. (ongoing)	Peanut (7)	30 months	At 4 months, no significant change in peanut-specific IgE, IgG, IgG4, or skin prick wheal diameter.
Oral			
Patriarca et al. [26]	Varied (24)	18 months	Desensitization was successful in 45 (77%) of 58 treatments.
Buchanan et al. [27]	Egg (7)	24 months	Four (57%) of 7 passed food challenge with 14.7 g of egg at conclusion of therapy; 2 passed second challenge 3-4 months later.
Staden et al. [28]	Cow's milk or egg (45)	11-59 months	Nine of 25 children (36%) showed tolerance in the treatment and in the control group, 7 of 20 children (35%) were tolerant.
Skripak et al. [29]	Cow's milk (19)	5-6 months	Eight out of 19 (42%) passed food challenge with 8 g of milk. Treatment subjects who underwent a posttreatment challenge had significant median increase in threshold dose.
Jones et al. [30]	Peanut (29)	18 months	Twenty-seven of 29 (93%) passed food challenge to 3.9 g of peanut.
Varshney et al. (ongoing)	Peanut (27)	1 year	All treatment subjects that have undergone food challenges have tolerated 5 g of peanut.

Adapted from Burks et al. Oral tolerance, food allergy, and immunotherapy: implications for future treatment. *J Allergy Clin Immunol*. 2008;121:1348.

### Heat Denatured Protein

High heat reduces the allergenicity of many food proteins by altering the conformation of heat-labile proteins and eliminating conformational epitopes[31]. Patients with persistent milk allergy possess higher detectable levels of IgE antibodies to linear epitopes from casein than patients who have achieved tolerance[32]. Lemon-Mule et al demonstrated that of 117 subjects with documented IgE mediated allergy to egg, 27 were heated egg reactive, 64 were heated egg tolerant, 23 were egg (heated and unheated) tolerant[33]. Thus, 70% of children with egg allergy were tolerant to heated egg. Those subjects who ingested heated egg after passing the egg challenge had significant decreases in their egg white SPTs (*P *< 0.001), increased ovalbumin and ovomucoid-specific IgG4 levels from baseline at 3, 6, and 12 months (*P *< 0.001), and decreased ovalbumin-specific IgE levels at 12 months (*P *< 0.001). Of note, the egg white and ovomucoid-specific IgE levels did not differ from baseline.

The same group looked at 100 milk allergic children and found that 68 tolerated extensively heated milk only, 23 reacted to heated milk, and 9 tolerated both heated and unheated milk[34]. Overall 78% of the children tolerated heated milk. The subjects who tolerated extensively heated milk, but reacted to unheated milk were placed on a diet containing baked milk products. Immunologically, these subjects at 3 months had smaller median milk SPT diameters (*P *= 0.001) and an increase in median casein-specific IgG4 concentrations (*P *= 0.005). There was no change seen in median casein and *β*-lactoglobulin-specific IgE, *β*-lactoglobulin-specific IgG4, or milk-specific IgE levels.

The implications from this study could mean that those heated-antigen tolerant patients may actually have an accelerated course to tolerance by ingesting the implicated heated food. This form of immunotherapy is still under investigation, but it may represent another avenue of treatment.

### Engineered Antigen

One approach to immunotherapy is to create engineered (mutated) proteins in which the IgE-binding epitopes are altered thereby decreasing the allergenicity whereas sparing the protein's ability to stimulate antigen-specific T cells. There have been several recombinant food proteins created[35-37]. One in vivo study using a murine model has demonstrated efficacy in preventing peanut induced anaphylaxis[37]. Peanut-allergic C3H/HeJ mice received heat-killed *Escherichia coli *producing engineered (mutated) Ara h1, 2, and 3 (HKE-MP123) rectally. The medium- and high-dose HKE-MP123-treated mice exhibited reduced symptom scores on subsequent peanut challenges for up to 10 weeks after treatment accompanied by a significant reduction of plasma histamine levels compared with sham-treated mice (*P *< 0.05 and .01, respectively). IgE levels were significantly lower in all HKE-MP123-treated groups (*P *< 0.001). Phase I clinical trials are presently ongoing.

### Peptide Immunotherapy

Small peptides cannot cross-link IgE bound to mast cells, so degranulation would not occur. Theoretically, a vaccine composed of overlapping small peptides that span the sequence of allergenic proteins could present epitopes to T cells without causing an allergic response. Although preliminary murine models of this strategy have shown promise, validating the stability and uniformity of a complex vaccine has proven to be difficult and has thus hindered development [11].

### Plasmid DNA

By creating vaccines that use bacterial plasmid DNA that encode an allergen, it is hypothetically possible to induce tolerance by the production of endogenously produced antigens that would not stimulate an allergic response. Oral administration of plasmid DNA complexed with chitosan, a natural biocompatible polysaccharide, resulted in the prevention of peanut allergy in AKR/J mice[38]. Mice receiving these DNA nanoparticles containing Ara h 2 produced secretory IgA and serum IgG2a and had reduced levels of IgE, plasma histamine and vascular leakage. However, a later study found that AKR/J, but not C3H/HeJ mice, were protected from peanut allergy using intramuscular injections of plasmid DNA encoding Ara h 2[39]. The different outcomes for these different strains of mice call in to question the effectiveness of this therapy in clinical trials.

### Allergen-Conjugated Immune Stimulatory Sequences

Another strategy employs the use of allergen determinants that augment T_H_1 responses via the innate immune system. Certain pathogen associated molecular patterns (PAMPs) can act as immune stimulatory sequences that enhance the T_H_1 response thus skewing the immune system away from a T_H_2 phenotype. Nguyen et al found that using a vaccine with the immunostimulatory sequence oligode-oxynucleotide on subsequently T_H_2-sensitized mice significantly reduced the risk of death after anaphylactic challenge[40]. This strategy may be effective in preventing allergy, but its value at treating ongoing food allergy in still in question.

## Allergen Nonspecific Immunotherapy

An alternative approach to food allergy is to more globally suppress the T_H_2 response rather than treating the protein specific reaction. For those patients with multiple food allergies, this could prove to be invaluable.

### Anti-IgE Therapy

Leung et al used the humanized monoclonal anti-IgE molecule TNX-901 in a double-blinded, placebo-controlled trial of peanut allergy[41]. Different doses of TNX-901 were given subcutaneously for 16 weeks to subjects with proven peanut allergy from baseline food challenge. The highest dose (450 mg) of TNX-901 significantly increased the threshold dose from 178 mg to 2.8 g (*P *< 0.001). Unfortunately, 25% of participants received no benefit and no biomarkers or parameters were identified that could distinguish responders from nonresponders.

A subsequent double-blinded, placebo-controlled study using another monoclonal anti-IgE molecule, omalizumab, in peanut allergic subjects for 20-22 weeks was discontinued because of safety concerns related to the pre-omalizumab challenges. Limited data showed possible increase in tolerance of omalizumab versus placebo treated subjects (44.4% vs 20% tolerated > 500 mg peanut protein). More studies are needed to elucidate this treatment modality.

### Chinese Herbal Medicine

A mixture of 9 traditional Chinese herbs, named Food Allergy Herbal Formulation-2 (FAHF-2), has shown promising results in a murine model of allergy[42-44]. Mice allergic to peanut treated with FAHF-2 for 7 weeks were challenged 1, 3, or 5 weeks posttherapy. After challenges, all sham-treated mice developed severe anaphylactic signs while no sign of anaphylactic reactions was observed in FAHF-2-treated mice. IgE levels were significantly reduced by FAHF-2 treatment and remained significantly lower as long as 5 weeks posttherapy[42]. In a follow up study, C3H/HeJ mice previously sensitized to peanut were treated with 7 weeks of FAHF-2 or sham treatment[43]. Mice were subsequently challenged 1 day posttherapy and 4 weeks posttherapy. All sham-treated mice showed anaphylactic symptoms on the subsequent challenges versus the FAHF-2-treated mice that showed no sign of anaphylactic reactions. Peanut-specific IgE levels in FAHF-2-treated mice also were reduced (4 weeks posttherapy, *P *< 0.001) whereas IgG2a levels were increased (4 weeks post-therapy, *P *< 0.001). More recently, the same group has now shown that the protective effect of 7 weeks of FAHF-2 on mice lasts more than 36 weeks[44]. They also found that the beneficial effect of FAHF-2 is mediated largely by elevated CD8^+ ^T-cell IFN-*γ *production. Phase I human trials are currently ongoing.

### Cytokine Therapy

It has been shown that the administration of a Lactococcus lactis transfected to secrete IL-10 prevented sensitization in a mouse model of food allergy[45]. IL-10 is a known suppressive regulatory cytokine. The administration of recombinant mouse IL-21 or an IL-21 expression plasmid suppressed anaphylaxis in mice previously sensitized to peanut[46]. IL-21 was shown to regulate systemic allergic reactions by inducing the transcriptional regulator Id2, which blocks B cell class switch recombination and IgE production. TGF-ß may also prove to be an effective immune modulator for food allergy. BALB/c mice treated orally with ovalbumin and TGF-*β *showed reduction of ovalbumin-specific IgE and IgG1 antibodies, T-cell reactivity, and immediate-type skin reactions when compared with the mice treated orally with ovalbumin alone[47]. Li et al demonstrated that an increase of TGF-*β *protein in BALB/c mouse intestinal tissue can be induced after oral administration of a TGF-*β *expressing DNA vector that was packed in chitosan nanoparticles[48]. A significant amelioration of ovalbumin-induced food allergy symptoms was also shown. Cytokine therapy holds a great potential, but it requires further investigation.

## Alternative Therapies

Other strategies targeting specific immune pathways have emerged in murine models that may hold promise. Blocking the interaction between dendritic cell TIM-4 and T cell TIM-1 abolished peanut extract specific T_H_2 polarization and allergy in the intestine of BALB/c mice[49]. Targeted inhibition of the high affinity IgE-receptor via a mouse Fc*ε*-Fc*γ *fusion protein suppressed the ability to sensitize mast cells in vitro[50]. A human homologue of this protein prevented anaphylaxis to Ascaris in sensitized monkeys. Zhu et al demonstrated that administration of a synthetic agonists of Toll-like receptor 9 (immune modulatory oligonucleotide) in a murine model had the capacity to switch peanut induced skewing away from a T_H_2 immune responses toward a T_H_1 response accompanied by reduced inflammation in the gastrointestinal tract and anaphylaxis in both prevention and treatment models[51]. Using a combination of allergen specific and nonspecific strategies are also under investigation. At Duke, we are beginning a clinical trial using concurrent anti-IgE therapy with peanut oral immunotherapy in the treatment of peanut allergy.

## Conclusion

The increasing prevalence of food allergy has brought to the forefront the lack of treatment options and the impact this disease has on patients, families, and the medical system. There is an urgent need for more safe and effective treatment options for these patients. Although genetic factors play a role, the rising incidence of food allergy over the past few decades must be explained by environmental variables. These variables represent opportunities for potential therapies for prevention or treatment of existing disease. As our knowledge of the formation and loss of tolerance grows, new immune pathways are emerging as possible targets of immunotherapy. Strategies manipulating these pathways are under investigation and provide hope for food allergy patients and their families.
